# Comparison of 30-day postoperative complications following sleeve gastrectomy vs. one-anastomosis gastric bypass

**DOI:** 10.1007/s00423-026-04137-9

**Published:** 2026-07-21

**Authors:** Michal Ness Kievsky, Arik Dahan, Carmil Azran

**Affiliations:** 1 Miriam and Sheldon G. Adelson School of Medicine at Ariel University, Ariel, Israel; 2 Department of Clinical Pharmacology, School of Pharmacy, Ben-Gurion University of the Negev, Beer-Sheva, 8410501 Israel; 3 Department of Medical Technologies, Maccabi Healthcare Services, Tel-Aviv, 6772168 Israel; 4 School of Pharmacy, Faculty of Medicine, The Hebrew University of Jerusalem, Jerusalem, 9112102 Israel

**Keywords:** Bariatric surgery, Sleeve gastrectomy, One anastomosis gastric bypass, Postoperative complications, Retrospective studies

## Abstract

**Background:**

Metabolic Bariatric Surgery (MBS) is highly effective in promoting weight loss and weight loss maintenance in patients with obesity. Worldwide, the predominant MBS was sleeve gastrectomy (SG), followed by the Roux-en-Y bypass, while One-anastomosis gastric bypass (OAGB, mini-gastric bypass) only accounted for 4.3% of the surgeries. Conversely, In Israel alone in 2020 OAGB accounted for 63.7% of all bariatric surgeries, followed by SG. This provides a genuine opportunity to compare postoperative complications amongst these bariatric procedures.

**Aim:**

In this retrospective study we reviewed medical records of 2765 patients who have undergone SG or OAGB in the Herzliya Medical Center (HMC) between the years 2018–2020, and compared 30-day postoperative complications between the two procedures.

**Methods:**

The 30-day timeframe was divided into two periods, the first consisting of hospitalization and included complications such as need for blood or plasma infusion during surgery, cardiorespiratory complications. The second period extended from discharge until the end of the 30th postoperative day and included complications such as surgical wound infection and other infections.

**Results:**

During hospitalization, 1.7% of SG patients had complications and 2.2% of OAGB patients had complications (*p* = 0.374). In the second period, 3.8% of SG patients and 4.1% of OAGB patients had complications (*p* = 0.907).

**Conclusion:**

These results indicate that there was no difference in the 30-day postoperative complication rate between these two types of bariatric procedures.

## Introduction

Obesity (BMI ≥ 30 kg/m^2^) is a common disorder, and its prevalence has increased markedly during the last three decades. According to the WHO, in 2022 there were more than 2.5 billion adults worldwide 18 or older that were overweight, 35.6% of them had obesity [1]. In a 2019 survey done by the Israeli Center for Disease Control, 56% of Israeli adults over the age of 21 are either overweight or obese[2].

Obesity is associated with many medical problems, such as cardiovascular diseases, diabetes, musculoskeletal disorders, obstructive sleep apnea, polycystic ovary syndrome (PCOS), some cancers and is a risk factor for SARS-CoV-2 poor prognosis [1].

Metabolic Bariatric Surgery (MBS) is highly effective in promoting weight loss and weight loss maintenance in patients with obesity [3]. Studies have shown that MBS can improve and even resolve many weight-related medical problems [4, 5].

MBS involves either reduction of stomach volume, metabolic effects, or a combination of these mechanisms.

Surgeries that reduce stomach volume such as adjustable gastric banding and sleeve gastrectomy. Adjustable gastric banding is the least invasive of the these surgeries, which limits food intake by placing a constricting ring around the fundus of the stomach. Sleeve gastrectomy is a restrictive method, with metabolic effects, in which most of the greater curvature is removed and the stomach’s size is reduced to about 25% of the original size. The gastrectomy is performed vertically, leaving the pyloric valve intact.

Procedures that combine both the reduction of stomach volume a short intestine and decrease in absorption mechanism are the Roux-en-Y bypass and the one-anastomosis gastric bypass procedures (OAGB). The OAGB is a modification of Mason’s loop gastric bypass; the stomach is divided to create a small pouch and is then anastomosed to the small intestine, about 200 cm distal to the ligament of Treitz [3, 4, 6]. This procedure is gaining popularity due to its simplicity and effectiveness in achieving significant weight loss and metabolic improvements.

MBS provides long-term health improvements such as body weight reduction, improvement in glycemic control and blood lipids [7].

**Fig. 1:**
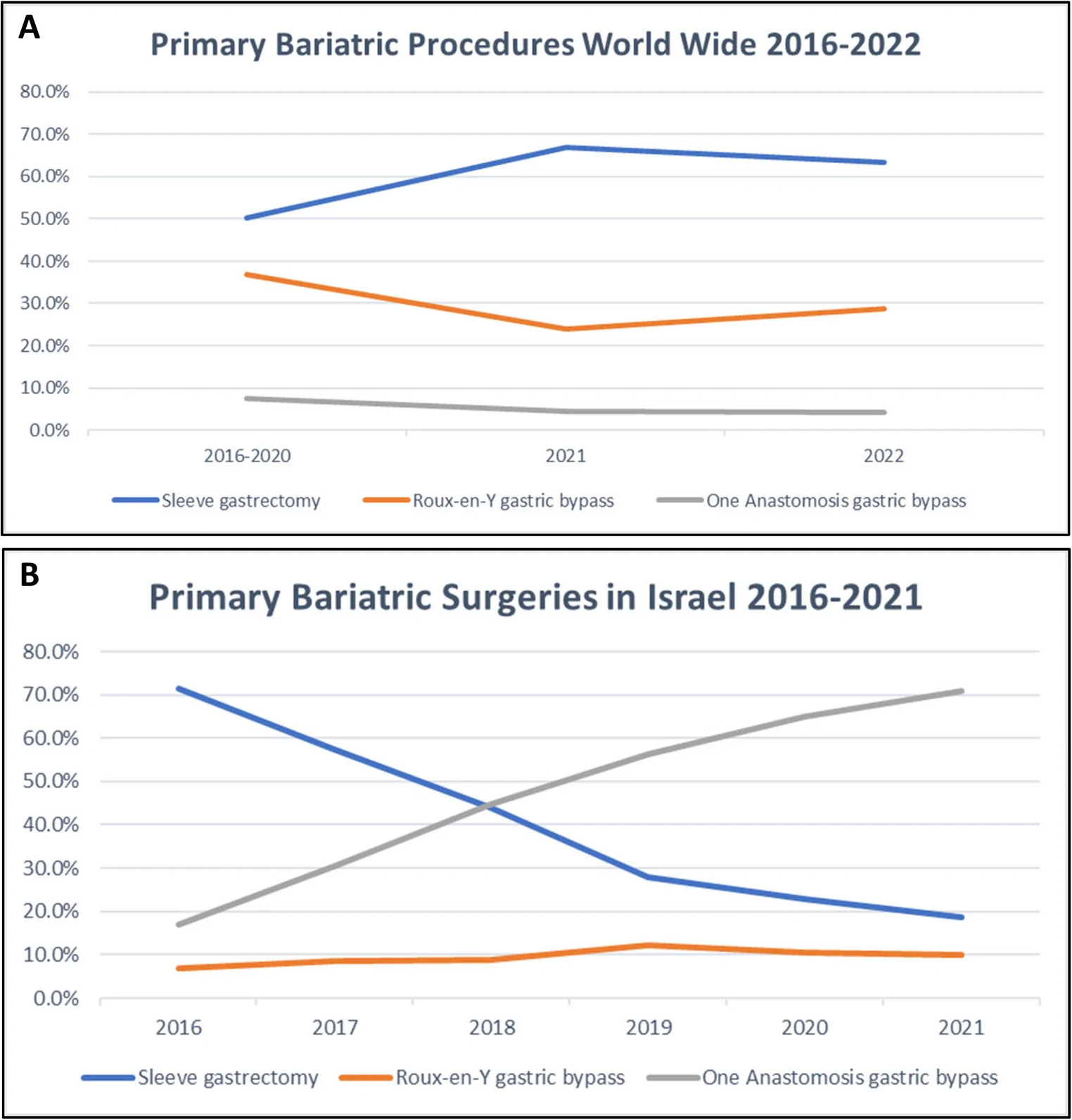
Primary type of bariatric surgeries worldwide (panel **A**) vs. primary type of bariatric surgeries in Israel (panel **B**) for the years 2016–2022. (**B**) shows the Israeli trend over the years where OAGB has become the popular MBS, surpassing sleeve gastrectomy, as opposed to the rest of the world shown in (**A**), showing sleeve gastrectomy as predominant MBS worldwide in the years 2016–2022. Data was taken from Israeli Society for Metabolic & Bariatric Surgery, National registry of bariatric surgery – ICDC and ISMBS and IFSO databases for the years 2016–2021 and 2016–2022, accordingly

According to the International Federation for the Surgery of Obesity and Metabolic Disorders (IFSO), in 2022 the predominant MBS worldwide was sleeve gastrectomy (60.4%), followed by the Roux-en-Y bypass (29.5%). The OAGB only accounted for 4.3% of the surgeries worldwide [8] (Fig. 1A).

However, as opposed to the rest of the world, in Israel in 2020 the predominant surgery was the OAGB (63.7%) followed by the sleeve gastrectomy (19.2%) [9]. In 2021, the OAGB surgery accounted for 68.2% of all bariatric surgeries, only to be followed by the sleeve gastrectomy (16.2%) and the Roux-en-Y gastric bypass (13.2%) [10]. (Fig. 1B).

According to the Israeli National Registry of Bariatric Surgery, in 2021, there were 8,192 bariatric surgeries performed on adults over the age of 18, 16.5% of which were revisional surgeries. The average age for MBS was 38.9 ± 12.0 with an average BMI of 41.4 [10].

As with any surgery, MBS occasionally has early and long postoperative complications. Common early general postoperative complications include anastomotic leak, wound infection, venous thromboembolism, cardiac complications, respiratory complications, internal bleeding, and sepsis [10]. Each surgery type carries its unique long term complications, due to the specific nature of the surgical procedure, such complications include anastomotic stenosis, balloon complications, band erosions, GERD, bile reflux and sleeve leaks [5, 11, 12].

Most data available of postoperative complications is obtained with sleeve gastrectomy making up as the majority of bariatric surgeries. The data available in Israel about OAGB provides a genuine opportunity to compare postoperative complications amongst the two types of bariatric surgeries, sleeve gastrectomy and OAGB. In this study we compared complications occurring within the first 30 days following sleeve gastrectomy and OAGB.

## Methods

### Study design

The study is a retrospective cohort designed to compare the incidence of complications 30 days following surgery between sleeve gastrectomy and OAGB. Sleeve gastrectomy and OAGB surgeries were performed by eight (8) highly experienced high-volume bariatric surgeons, following standardized techniques, minimizing surgeon-level variability. The study included adult patients (age ≥ 18 years) who underwent either sleeve gastrectomy or OAGB in the Herzliya Medical Center between January 2018 – December 2020, and responded to the 30-day telephonic follow-up.

Data was collected from electronic records of patients who underwent sleeve gastrectomy or OAGB at the Herzliya Medical Center, a private hospital, leading in bariatric surgeries, between the years January 2018 through December 2020. The study was approved by the Helsinki Committee of Herzliya Medical Center (HMC) approval #0015–21-HMC.

For each participant demographic and clinical data were extracted from the Herzliya Medical Center electronic records, including age, sex, body mass index (BMI), associated medical problems, previous MBS, type of current MBS, length of hospital stay and post operative complications during hospitalization.

The study had two parts to it, the first was data from hospitalization collected from medical databases. Pre-surgical and hospitalization data were extracted from the electronic records of the Herzliya Medical Center. The second part included patients who were surveyed by telephone during the period between discharge and the 30th day postoperative period. This data was extracted from Monitor Care, an extract of a scripted postoperative survey, gathered via a routine phone call, done periodically after surgery during the 30-day period. The survey was validated, had a unified standard question form with a dedicated space for comments.

.

Complication rates were measured in two time frames: the first consisted of the hospitalization period, and the second extending from the day of discharge until the end of the 30th postoperative day.

The complications for the hospitalization period included immediate complications: need for blood or plasma infusion during surgery, vein thrombotic events, wound infection, anastomosis leak or abscess, cardiorespiratory complications (pulmonary embolism, pneumonia and pulmonary infiltrates), and sepsis. Complications after discharge included surgical wound infection (redness, swelling or fever, secretion from wound), and other infections, bleeding, or antibiotic treatment due to suspicion of infection.

### Statistical analysis

Summary statistics for patient demographics and baseline characteristics were calculated for both the sleeve gastrectomy and OAGB groups. Continuous variables were presented as means with standard deviations (SD), while categorical variables were reported as percentages.

Logistic regressions were used to compare the two surgeries to see if one surgery is more prone to a specific complication. Multivariable logistic regression analyses were performed to evaluate the association between surgery type and postoperative complications while adjusting for potential confounders including age, BMI, smoking status, previous bariatric surgery, previous abdominal surgery, hypertension, diabetes, GERD, and additional baseline covariates.

All statistical analysis were conducted using R software version 4.2.0, and a two-sided p-value < 0.05 was considered statistically significant.

## Results

A total of 2765 patients were included in the study. Sleeve gastrectomy was performed on 829 patients (30%) and 1936 (70%) patients had OAGB. 76% (2102) of patients were female and 24% were male (663 patients). The characteristics of these 2765 patients are summarized in Table 1.

**Table 1:** Descriptive statistics of patients according to type of surgery

	Sleeve gastrectomy (*N* = 829)	One anastomosis gastric bypass (*N* = 1936)	*P*
Age			
Mean (SD)	37.6 (12.3)	38.4 (10.8)	0.102
Median [Min, Max]	36.3 [18.0, 71.2]	37.9 [18.1, 76.6]	
BMI			
Mean (SD)	41.7 (4.85)	41.8 (5.09)	0.796
Sex			< 0.001
Male	236 (28.5%)	427 (22.1%)	
Female	593 (71.5%)	1509 (77.9%)	
Smoker	224 (27.0%)	407 (21.0%)	< 0.001
Previous Bariatric Surgery	39 (4.7%)	326 (16.8%)	< 0.001
Previous stomach surgery	25 (3.0%)	61 (3.2%)	0.946
previous abdominal surgery	265 (32.0%)	663 (34.2%)	0.263
Hypertension	117 (14.1%)	274 (14.2%)	1
Diabetes	81 (9.8%)	197 (10.2%)	0.798
Sleep apnea	91 (11.0%)	150 (7.7%)	0.00726
Ischemic heart disease	8 (1.0%)	13 (0.7%)	0.565
Abnormal menstrual cycle	79 (9.5%)	148 (7.6%)	0.114
Fertility issues	30 (3.6%)	72 (3.7%)	0.986
Fatty Liver Disease	676 (81.5%)	1531 (79.1%)	0.154
Dyslipidemia	140 (16.9%)	336 (17.4%)	0.808
Thromboembolic event	14 (1.7%)	18 (0.9%)	0.13
Pseudotumor cerebri	6 (0.7%)	8 (0.4%)	0.446
Stroke	8 (1.0%)	19 (1.0%)	1
Asthma	37 (4.5%)	81 (4.2%)	0.818

The average age was 37.6y (SD = 12.3) for sleeve gastrectomy and 38.4y (SD = 10.8) for OAGB (*p* = 0.102). The mean BMI was 41.7 kg/m^2^ (SD = 4.85) for sleeve gastrectomy and 41.8 kg/m^2^ (SD = 5.09) for OAGB (*p* = 0.796).

The most prevalent associated medical problemsfor patients undergoing sleeve gastrectomy were fatty liver disease (81.5%), dyslipidemia (16.9%), hypertension (14.1%), and sleep apnea (11%). The most prevalent associated medical problem for patients undergoing OAGB were fatty liver disease (79.1%), dyslipidemia (17.4%), hypertension (14.2%) and diabetes (10.2%). GERD was more prevalent in the OAGB group (*p* = 0.017), while depression/anxiety were more prevalent in the sleeve gastrectomy group (*p* = 0.004).

### Complications from surgery until discharge

Mean hospitalization length was 2.11 days (SD = 0.47) for sleeve gastrectomy and 2 days.

Complications occurring between surgery until discharge (Table 2) were observed in 14/829 (1.7%) patients that underwent sleeve gastrectomy, and 42/1936 (2.2%)tof the patients who underwent the OAGB surgery (*p* = 0.374).

**Table 2:** Statistical analysis of complications that occurred in the period between surgery until discharge

Complication	Sleeve gastrectomy (*n* = 829)	One anastomosis gastric bypass (*n* = 1936)	OR	*P*-value
Received blood or plasma during surgery (%)	7 (0.8)	24 (1.2)	1.496	0.363
Life threatening complication (%)	2 (0.2)	1 (0.1)	0.282	0.323
Anastomosis leak or abscess (%)	1 (0.1)	3 (0.2)	1.263	0.865
Wound infection (%)	0 (0)	0 (0)	1	1
DVT (%)	1 (0.1)	0 (0)	0	0.997
Cardiorespiratory complication (%)	4 (0.5)	1 (0.1)	0.127	0.068
Sepsis (%)	1 (0.1)	0 (0)	0	0.997
Bleeding (%)	6 (0.7)	30 (1.5)	2.276	0.072
Invasive procedure during hospitalization (%)	5 (0.6)	9 (0.5)	0.876	0.815
ICU (%)	4 (0.5)	9 (0.5)	0.951	0.936
Total (%)	14 (1.7)	42 (2.2)	1.331	0.374

Complications were retrospectively graded according to the Clavien-Dindo classification (Table 3).

**Table 3:** Clavien-Dindo classification for complications that occurred in the period between surgery until discharge

Complication	Grade I	Grade II	Grade III a	Grade III b	Grade IV a	Grade IV b	Grade V
Received blood or plasma during surgery (%)							
Sleeve gastrectomy	5 (0.6)	0 (0)	0 (0)	0 (0)	2 (0.24)	0 (0)	0 (0)
One Anastomosis Gastric Bypass	18 (0.93)	0 (0)	0 (0)	0 (0)	6 (0.31)	0 (0)	0 (0)
Life threatning complication (%)							
Sleeve gastrectomy	0 (0)	0 (0)	0 (0)	0 (0)	2 (0.24)	0 (0)	0 (0)
One Anastomosis Gastric Bypass	0 (0)	0 (0)	0 (0)	0 (0)	1 (0.05)	0 (0)	0 (0)
Anastomosis leak or abscess (%)							
Sleeve gastrectomy	0 (0)	0 (0)	0 (0)	0 (0)	1 (0.12)	0 (0)	0 (0)
One Anastomosis Gastric Bypass	0 (0)	0 (0)	0 (0)	2 (0.1)	1 (0.05)	0 (0)	0 (0)
Wound infection (%)							
Sleeve gastrectomy	0 (0)	0 (0)	0 (0)	0 (0)	0 (0)	0 (0)	0 (0)
One Anastomosis Gastric Bypass	0 (0)	0 (0)	0 (0)	0 (0)	0 (0)	0 (0)	0 (0)
DVT (%)							
Sleeve gastrectomy	0 (0)	0 (0)	0 (0)	0 (0)	1 (0.12)	0 (0)	0 (0)
One Anastomosis Gastric Bypass	0 (0)	0 (0)	0 (0)	0 (0)	0 (0)	0 (0)	0 (0)
Cardiorespiratory complication (%)							
Sleeve gastrectomy	1 (0.12)	0 (0)	0 (0)	0 (0)	2 (0.24)	0 (0)	0 (0)
One Anastomosis Gastric Bypass	1 (0.05)	0 (0)	0 (0)	0 (0)	0 (0)	0 (0)	0 (0)
Sepsis (%)							
Sleeve gastrectomy	0 (0)	0 (0)	0 (0)	0 (0)	1 (0.12)	0 (0)	0 (0)
One Anastomosis Gastric Bypass	0 (0)	0 (0)	0 (0)	0 (0)	0 (0)	0 (0)	0 (0)
Bleeding (%)							
Sleeve gastrectomy	4 (0.48)	0 (0)	0 (0)	0 (0)	2 (0.24)	0 (0)	0 (0)
One Anastomosis Gastric Bypass	25 (1.29)	0 (0)	0 (0)	0 (0)	5 (0.26)	0 (0)	0 (0)
Invasive procedure during hospitilization (%)							
Sleeve gastrectomy	0 (0)	0 (0)	0 (0)	3 (0.36)	2 (0.24)	0 (0)	0 (0)
One Anastomosis Gastric Bypass	0 (0)	0 (0)	0 (0)	6 (0.31)	3 (0.15)	0 (0)	0 (0)
ICU (%)							
Sleeve gastrectomy	0 (0)	0 (0)	0 (0)	0 (0)	4 (0.48)	0 (0)	0 (0)
One Anastomosis Gastric Bypass	0 (0)	0 (0)	0 (0)	0 (0)	9 (0.46)	0 (0)	0 (0)

The main complications were bleeding 6 (0.7%) patients in the sleeve gastrectomy group and 30 (1.5%) of patients who underwent OAGB (*p* = 0.072). Subsequently the need for blood or plasma during surgery was 7 (0.8%) patients in the sleeve gastrectomy group and 24 (1.2%) patients who underwent OAGB (*p* = 0.363).

Cardiorespiratory and bleeding complications, during hospitalization, were both close to significance, with sleeve gastrectomy being more prone to cardiorespiratory complications than OAGB (OR = 0.127, *p* = 0.068) and in OAGB patients there was a trend towards higher bleeding rates than patients who underwent sleeve gastrectomy (OR = 2.276, *p* = 0.072). However, it was not statistically significant.

### Complications from discharge until the 30th postoperative day

The post-discharge survey was fully answered by 859 patients, 345 (40.2%) for sleeve gastrectomy, and 514 (59.8%) for OAGB. The characteristics of these 859 patients are summarized in Table 4.

**Table 4:** Descriptive statistics of patients completing the second survey time frame (discharge to 30th days post-surgery) according to type of surgery

	Sleeve gastrectomy (*N* = 345)	One anastomosis gastric bypass (*N* = 514)	*P*
Age			
Mean (SD)	37.5 (11.9)	39.7 (11.2)	0.102
BMI			
Mean (SD)	41.0 (5.0)	41.7 (5.2)	0.796
Sex			< 0.001
Male	90 (26.1%)	149 (28.9%)	
Female	255 (73.9%)	365 (71.1%)	
Smoker	97 (28.1%)	112 (21.7%)	0.018
Previous Bariatric Surgery	14 (4.0%)	106 (20.6%)	< 0.001
Hypertension	43 (12.4%)	88 (17.1%)	< 0.001
Diabetes	37 (9.8%)	62 (12.0%)	0.021
Sleep apnea	34 (9.8%)	33 (6.4%)	0.045
Ischemic heart disease	4 (1.1%)	6 (1.1%)	0.754
Abnormal menstrual cycle	38 (11.0%)	34 (6.6%)	0.048
Fertility issues	15 (4.3%)	9 (1.7%)	0.075
Fatty Liver Disease	280 (81.1%)	415 (80.7%)	0.014
Dyslipidemia	58 (16.8%)	92 (17.8%)	0.024
Thromboembolic event	6 (1.7%)	2 (0.4%)	0.089
Pseudotumor cerebri	0	3 (0.5%)	0.013
Stroke	1 (0.3%)	6 (1.1%)	0.074
Asthma	20 (5.7%)	19 (3.6%)	0.032
COPD	3 (0.8%)	2 (0.4%)	0.679
GERD	15 (4.3%)	45 (8.7%)	0.017
Depression/Anxiety	24 (6.9%)	23 (4.4%)	0.071

Complications between discharge until the 30th postoperative day (Table 5) were observed in 13/345 (3.8%) of the patients who underwent sleeve gastrectomy, and 21/514 (4.1%) of the patients who underwent the OAGB surgery (*p* = 0.907).

**Table 5:** Statistical analysis of the complications from discharge until the 30th postoperative day

Complication	Sleeve gastrectomy (*n* = 346)	One anastomosis gastric bypass (*n* = 516)	OR	*P*-value
Pain (%)	167 (48.4)	228 (44.4)	0.6224	0.258
Redness, swollen or fever (%)	1 (0.3)	0 (0)	0	0.999
Leak (%)	0 (0)	3 (0.6)	ND	0.998
Infection (%)	3 (0.9)	10 (1.9)	2.213	0.258
Secretions from Wound (%)	4 (1.2)	5 (1.0)	0.649	0.574
Secondary hospitalization (%)	9 (2.6)	11 (2.1)	0.657	0.4
Fever (%)	7 (2.0)	9 (1.8)	0.86	0.999
Bleeding (%)	0 (0)	1 (0.2)	ND	0.247
Antibiotic treatment (%)	7 (2.0)	11 (2.1)	0.964	0.945
Pus (%)	1 (0.3)	3 (0.6)	2.354	0.501
Total (%)	13 (3.8)	21 (4.1)	0.956	0.907

Complications were retrospectively graded according to the Clavien-Dindo classification (Table 6).

**Table 6:** Clavien-Dindo classification for complications that occurred from discharge until the 30th postoperative day

Complication	Grade I	Grade II	Grade III a	Grade III b	Grade IV a	Grade IV b	Grade V
Pain (%)							
Sleeve gastrectomy	162 (47)	0 (0)	5 (1.45)	0 (0)	0 (0)	0 (0)	0 (0)
One Anastomosis Gastric Bypass	224 (43.5)	0 (0)	4 (0.77)	0 (0)	0 (0)	0 (0)	0 (0)
Redness, swollen or fever (%)							
Sleeve gastrectomy	0 (0)	0 (0)	1 (0.29)	0 (0)	0 (0)	0 (0)	0 (0)
One Anastomosis Gastric Bypass	0 (0)	0 (0)	0 (0)	0 (0)	0 (0)	0 (0)	0 (0)
Leak (%)							
Sleeve gastrectomy	0 (0)	0 (0)	0 (0)	0 (0)	0 (0)	0 (0)	0 (0)
One Anastomosis Gastric Bypass	0 (0)	0 (0)	0 (0)	3 (0.58)	0 (0)	0 (0)	0 (0)
Infection (%)							
Sleeve gastrectomy	0 (0)	0 (0)	3 (0.87)	0 (0)	0 (0)	0 (0)	0 (0)
One Anastomosis Gastric Bypass	6 (1.16)	0 (0)	2 (0.39)	2 (0.39)	0 (0)	0 (0)	0 (0)
Secretions from Wound (%)							
Sleeve gastrectomy	3 (0.87)	0 (0)	1 (0.29)	0 (0)	0 (0)	0 (0)	0 (0)
One Anastomosis Gastric Bypass	5 (0.97)	0 (0)	0 (0)	0 (0)	0 (0)	0 (0)	0 (0)
Secondary hosptilization (%)							
Sleeve gastrectomy	0 (0)	0 (0)	9 (2.6)	0 (0)	0 (0)	0 (0)	0 (0)
One Anastomosis Gastric Bypass	0 (0)	0 (0)	8 (1.55)	3 (0.58)	0 (0)	0 (0)	0 (0)
Fever (%)							
Sleeve gastrectomy	4 (1.16)	0 (0)	3 (0.87)	0 (0)	0 (0)	0 (0)	0 (0)
One Anastomosis Gastric Bypass	6 (1.17)	0 (0)	1 (1.19)	2 (0.39)	0 (0)	0 (0)	0 (0)
Bleeding (%)							
Sleeve gastrectomy	0 (0)	0 (0)	0 (0)	0 (0)	0 (0)	0 (0)	0 (0)
One Anastomosis Gastric Bypass	0 (0)	0 (0)	1 (1.19)	0 (0)	0 (0)	0 (0)	0 (0)
Antibiotic treatment (%)							
Sleeve gastrectomy	3 (0.87)	0 (0)	4 (1.16)	0 (0)	0 (0)	0 (0)	0 (0)
One Anastomosis Gastric Bypass	7 (1.36)	0 (0)	2 (0.39)	2 (0.39)	0 (0)	0 (0)	0 (0)
Pus (%)							
Sleeve gastrectomy	0 (0)	0 (0)	1 (0.29)	0 (0)	0 (0)	0 (0)	0 (0)
One Anastomosis Gastric Bypass	3 (0.58)	0 (0)	0 (0)	0 (0)	0 (0)	0 (0)	0 (0)

Patients with clinical signs of infection were present in both groups. For the sleeve gastrectomy, 7/345 (2%) patients had a fever, 1/345 (0.3%) patients experienced pus secretion from the wound. Three patients out of 345 (0.9%) who underwent sleeve gastrectomy were diagnosed with an infection. Seven out of 345 (2%) patients were treated with antibiotics. For the OAGB, 9/514 (1.8%) of the patients had a fever, 3/514 (0.6%) experienced pus secretion from the wound. Ten out of 514 (1.9%) patients who underwent OAGB were diagnosed with an infection and 11/514 (2.1%) of the patients were treated with antibiotics.

Secondary hospitalization was needed for 9/345 (2.6%) patients who underwent sleeve gastrectomy and 11/514 (2.1%) patients who underwent OAGB (*p* = 0.4).

Adjusted odds ratios (ORs) with 95% confidence intervals were calculated. Logistic regression analysis revealed that there was no statistically significant difference between the rate of complications between the two types of MBS. This is true for both complications during hospitalization (Table 2) and for complications from discharge until the end of the 30th postoperative day (Table 6).

## Discussion

Sleeve gastrectomy and OAGB are two popular bariatric surgeries with OAGB having a better long-term outcome achieving better weight loss and improvement of the associated medical problems [8, 13, 14].

Sleeve gastrectomy involves reduction of the stomach size, while OAGB involves both reduction of the stomach size and its connection to a distal part of the small intestine. Given the nature of these surgeries, it would be expected that OAGB would have more short-term complications, such as leaks and bleeding.

In our study we found that there is no difference in the complication rate during the 30 postoperative days. In both time periods we studied, the first including the hospitalization and second from the time of discharge until the end of the 30th postoperative day, there was no significant difference between the two types of surgery modalities.

In our study we found that there is no difference in the complication rate during the 30 postoperative days. In both time periods we studied, the first including the hospitalization and second from the time of discharge until the end of the 30th postoperative day, there was no significant difference between the two types of surgery modalities.

While long term complications for these procedures include anemia, vitamin and mineral deficiencies for OAGB and GERD for sleeve gastrectomy [14–16], w this study confirms that there is no significant difference in the short-term complications between both surgeries [17–21]. Our results are consistent with a meta-analysis of 10 studies that showed no difference in complications between sleeve gastrectomy and OAGB (odds ratio 0.85, 95% CI 0.59–1.24, *P* = 0.4) and with a study done on 341 cases that found comparable overall complications between patients who underwent sleeve gastrectomy and OAGB (6.7% versus 5.0%, *P*= 0.43) [22, 23].

The guidelines don’t have criteria for choosing a specific MBS, but rather describe who is a good candidate for MBS “bariatric surgery should be recommended for patients with a BMI greater than 35 kg/m^2^ regardless of the associated medical problems and for patients with a BMI between 30–34.9 kg/m^2^with a metabolic disease” [24]. There are relative contraindications for sleeve gastrectomy, such as GERD and a pre-existing large hiatal hernia [25]. Given these facts, we assume that there was an appropriate selection of surgery for each patient in our study. It should be noted that usually OAGB is suggested as primary surgery for patients with BMI > 50 kg/m^2 ^[26], in our study, this was not a criteria and patients with lower BMI did undergo OAGB as primary surgery.

Currently guidelines do not include a criterion for choosing a specific MBS, but rather describe who is a good candidate for MBS [24]. Our results show that both sleeve gastrectomy and OAGB have similar complication rates in the short term, giving the patient an option to choose the surgery based on long term outcomes, personal preference, and surgeon skills.

In our study we compared sleeve gastrectomy and OAGB. OAGB is a modification of the Roux-en-Y surgery, the second most popular MBS worldwide, therefore short-term complications for both surgeries should be compared in future studies. Currently the suggestion for OAGB as a primary surgery is a BMI greater than 50 kg/m^2 ^[26]. It should be further studied if OAGB is an appropriate primary surgery for candidates for MBS with a lower BMI.

It should be noted that since this study was a retrospective cohort, we had no influence regarding patient selection for each surgery. In addition, this study only included complications in the first 30 days following surgery. Moreover, while electronic medical records were available for all patients, the survey that was done after discharge required patient cooperation, which led to the significant dropping between the first and second survey time frames. The higher proportion of revisional procedures in the OAGB group may represent a potential confounding factor and should be considered when interpreting the results. Only patients that answered the survey fully were included. Grading severity using the Clavien-Dindo classification, the standard for reporting surgical complications, was done retrospectively. 

## Conclusion

During the 30 postoperative days there was no statistically significant difference in the rate of complications between sleeve gastrectomy and OAGB. Patient preferences, surgical anatomy, patient co-morbidities and long-term complication risk should be used to decide which surgery is the best procedure for the patient.

## Data Availability

All data generated or analyzed during this study are included in this article. Further enquiries can be directed to the corresponding author.
